# A High-Density Genome-Wide Association Screen of Sporadic ALS in US Veterans

**DOI:** 10.1371/journal.pone.0032768

**Published:** 2012-03-28

**Authors:** Lydia Coulter Kwee, Yutao Liu, Carol Haynes, Jason R. Gibson, Annjanette Stone, Steven A. Schichman, Freya Kamel, Lorene M. Nelson, Barbara Topol, Stephen K. Van Den Eeden, Caroline M. Tanner, Merit E. Cudkowicz, Daniela L. Grasso, Robert Lawson, Sumitra Muralidhar, Eugene Z. Oddone, Silke Schmidt, Michael A. Hauser

**Affiliations:** 1 Epidemiology Research and Information Center, Durham Veterans Affairs Medical Center, Durham, North Carolina, United States of America; 2 Center for Human Genetics, Duke University Medical Center, Durham, North Carolina, United States of America; 3 Department of Medicine, Duke University Medical Center, Durham, North Carolina, United States of America; 4 Pathology and Laboratory Medicine Service and Research Service, Central Arkansas Veterans Healthcare System, Little Rock, Arkansas, United States of America; 5 Department of Pathology, University of Arkansas for Medical Sciences, Little Rock, Arkansas, United States of America; 6 Epidemiology Branch, National Institute of Environmental Health Sciences, Research Triangle Park, North Carolina, United States of America; 7 Division of Epidemiology, Department of Health Research and Policy, Stanford University School of Medicine, Stanford, California, United States of America; 8 Division of Research, Kaiser Permanente Northern California, Oakland, California, United States of America; 9 The Parkinson's Institute, Sunnyvale, California, United States of America; 10 Massachusetts General Hospital, Neurology Clinical Trial Unit, Harvard Medical School, Boston, Massachusetts; 11 Office of Research and Development, Department of Veterans Affairs, Washington, District of Columbia, United States of America; National Institute of Health, United States of America

## Abstract

Following reports of an increased incidence of amyotrophic lateral sclerosis (ALS) in U.S. veterans, we have conducted a high-density genome-wide association study (GWAS) of ALS outcome and survival time in a sample of U.S. veterans. We tested ∼1.3 million single nucleotide polymorphisms (SNPs) for association with ALS outcome in 442 incident Caucasian veteran cases diagnosed with definite or probable ALS and 348 Caucasian veteran controls. To increase power, we also included genotypes from 5909 publicly-available non-veteran controls in the analysis. In the survival analysis, we tested for association between SNPs and post-diagnosis survival time in 639 Caucasian veteran cases with definite or probable ALS. After this discovery phase, we performed follow-up genotyping of 299 SNPs in an independent replication sample of Caucasian veterans and non-veterans (ALS outcome: 183 cases and 961 controls; survival: 118 cases). Although no SNPs reached genome-wide significance in the discovery phase for either phenotype, three SNPs were statistically significant in the replication analysis of ALS outcome: rs6080539 (177 kb from *PCSK2*), rs7000234 (4 kb from *ZNF704*), and rs3113494 (13 kb from *LOC100506746*). Two SNPs located in genes that were implicated by previous GWA studies of ALS were marginally significant in the pooled analysis of discovery and replication samples: rs17174381 in *DPP6* (p = 4.4×10^−4^) and rs6985069 near *ELP3* (p = 4.8×10^−4^). Our results underscore the difficulty of identifying and convincingly replicating genetic associations with a rare and genetically heterogeneous disorder such as ALS, and suggest that common SNPs are unlikely to account for a substantial proportion of patients affected by this devastating disorder.

## Introduction

Amyotrophic lateral sclerosis (ALS) is a fatal disease characterized by motor neuron degeneration, which leads to muscle atrophy and paralysis. Typically, this progressive muscle wasting results in death from respiratory failure within 2–4 years after onset of symptoms. ALS is the most common adult-onset motor neuron disease, with an incidence of 2–3 cases per 100,000 person-years [1], but its prevalence is low due to the poor prognosis associated with disease.

The majority of ALS cases are sporadic; approximately 5% of cases show a family history of disease [2]. Although several causative genes have been identified in familial ALS, the genetic contributors to sporadic ALS have been more difficult to identify. Genome-wide association studies of sporadic ALS have implicated a number of genes or regions (*DPP6*, *ITPR2*, *UNC13A*, *FGGY*, *ELP3*, *KIFAP3*, 9p21.2) [3]–[11], but replication of these findings in independent populations has proven difficult [12]–[16]. A possible exception is the 9p21.2 region, which was recently replicated by two large independent studies and seems to be important in both familial and sporadic ALS [17], [18].

Environmental exposures also appear to play a role in ALS incidence. Some of the reported associations include cigarette smoking [19], [20], head injury [21]–[23], exposure to lead or other heavy metals [24]–[27], exposure to pesticides [28], [29], and physical activity [30], [31], although subsequent independent studies have reported mixed results. Additionally, an increased incidence of ALS has been reported both specifically for US veterans of the Persian Gulf War [32]–[34] and more generally for all US veterans [35]. The nature of the relationship between military service and ALS merits further investigation into the possible aspects of military service (environmental exposures, deployment exposures, lifestyle behaviors) that may confer an increased risk of ALS.

Here, we describe a genome-wide association study (GWAS) performed in a population of US veterans. To our knowledge, this is the first genome-wide study designed to identify genetic factors that may contribute to ALS in a veteran population. Genotypes were obtained using two different arrays, which generated the highest density of single-nucleotide polymorphisms (SNPs) of all ALS GWA studies published to date. Additionally, the existence of overlapping probes on the two arrays allowed us to evaluate genotype concordance between the platforms and to reduce genotyping errors. In order to evaluate genetic factors associated with developing sporadic ALS as well as survival time after diagnosis of ALS, we performed both a case-control analysis and a survival analysis. Following the discovery phase of the study, we genotyped potentially-interesting SNPs in an independent replication study consisting of both veterans and non-veterans.

## Methods

### Ethics statement

This study was conducted in accordance with the Declaration of Helsinki of the World Medical Association. All study participants provided written informed consent. The research was reviewed and approved by the Institutional Review Boards from all participating institutions, including the Institutional Review Boards of the Durham VA and Duke University Medical Centers, the Parkinson's Institute, National Institutes of Health, National Institute of Environmental Health Sciences, University of Iowa, Massachusetts General Hospital, New England Medical Center, Brigham and Women's Hospital, Social and Scientific Systems, Inc., Battelle, Inc., Survey Research Associates, CODA, and the Human Subject Committees of the Kaiser Foundation Research Institute and Stanford University.

### Participants

Case samples for this study were obtained from the National Registry of Veterans with ALS, which enrolled 2121 US veterans between April 2003 and September 2007. We refer to the veteran cases here as “NRVA cases.” Methods for recruitment, medical record review, and enrollment into the Registry have been described in detail elsewhere [36]. Briefly, cases were actively recruited through periodic searches of Veterans Administration (VA) inpatient and outpatient databases for ICD-9 (International Classification of Diseases, 9th Revision, Clinical Modification) diagnoses of motor neuron disease. Passive recruitment methods included the distribution of study brochures and mailings to ALS specialty clinics and neurologists, along with links on ALS-related websites. Following informed consent and enrollment, neurologists reviewed each case's medical records to confirm a diagnosis of definite, probable or possible ALS according to the original El Escorial criteria [37], [38]. Patients were also enrolled in the Registry if they had “suspected ALS” according to the criteria, which included a diagnosis of progressive muscular atrophy (PMA), primary lateral sclerosis (PLS) or progressive bulbar palsy (PBP). A subset of enrollees (n = 1173) consented to participate in the registry-affiliated DNA bank and donated DNA via blood (84.6%) or mouthwash (15.4%), and 1163 of these samples were available for genotyping in this study. Registry enrollees were contacted for follow-up telephone interviews every 6 months from enrollment until September 30, 2009; the ALS Functional Rating Scale (ALSFRS-R) was administered at each of the follow-up interviews.

The veteran controls described here were enrolled in the “Genes and Environmental Exposures in Veterans with ALS” (GENEVA) study. As described previously [39], controls were identified from a database of US veterans maintained by the Veterans Benefit Administration and recruited via mailings and telephone calls. We refer to these controls as “GENEVA controls.” Controls were frequency-matched to cases on age (in 5-year intervals), gender, and use of the VA for health care (prior to diagnosis, for cases). Controls passed a telephone screener to confirm the absence of ALS and other neurological diseases. All of the GENEVA controls were administered telephone interviews about environmental exposures, as was a subset of the sampled NRVA cases (57%). Controls were also asked to donate a saliva sample for DNA extraction and genotyping; 411 control samples were genotyped in the discovery phase of this study. To improve the statistical power of our discovery GWAS, we also made use of approximately 6,000 publicly-available control genotypes generated by the same two high-density chips and distributed by the Wellcome Trust Case Control Consortium (WTCCC2) (http://www.wtccc.org.uk/ccc2/) [40]. Of these controls, 51.6% were recruited from the 1958 British birth cohort; the remainder comprised blood donors from the UK Blood Service Collection. As previously demonstrated [41], publicly available controls are well suited for inclusion into the analysis of a rare disease like ALS, because they have a very low probability of misclassification.

The replication phase of this analysis included 490 additional controls ascertained through the GENEVA study and 20 NRVA cases along with samples from the Northeast ALS Consortium (52 cases and 11 controls, contributed by MEC); the Agricultural Health Study (312 controls, contributed by FK); a New England study of ALS (108 cases and 38 controls, contributed by FK); and the Genetics and Epidemiology of Motor Neuron disease (GEM) study (100 cases and 308 controls, contributed by LMN). Details of sample ascertainment for each of these studies are given in Methods S1.

### Laboratory methods

For NRVA case samples, DNA was obtained from peripheral blood or from buccal cells. Blood was collected in 10 ml EDTA blood tubes. The blood was centrifuged for 15 min at 1850× g at 4°C; the plasma was removed and saved. The remaining buffy coat and red cells were transferred to a tube for DNA extraction. Buccal cells were collected in 10 ml original Mint Scope Mouthwash (Proctor & Gamble). The samples were centrifuged for 10 min at 2000× g and the supernatant discarded. DNA from both blood and buccal cells was extracted on an Autopure instrument using Puregene reagents (Qiagen, Valencia, CA). A subset of samples (1 blood; 16 buccal) did not have sufficient DNA available for genotyping. For these samples, DNA amplification was performed using the REPLI-g DNA amplification kit (Qiagen, Valencia, CA) according to the manufacturer's directions. Samples (20–50 ng) were amplified for 16 h and then purified by alcohol precipitation. For veteran control samples, DNA was extracted from Oragene samples (DNA Genotek, Inc.) using the Autopure LX following the manufacturer's recommended protocol.

### Genotyping and quality control

The discovery samples were genotyped on the Affymetrix Genome-Wide Human SNP Array 6.0 at Affymetrix, Inc. (Santa Clara, CA) and the Illumina Human1M-Duo genotyping array at the VA Pharmacogenomics Analysis Laboratory (Little Rock, AR). After calling the genotypes (see Methods S2 for a detailed description of the genotype-calling methods), we applied the following set of quality control (QC) filters to the autosomal SNPs on each array independently. We excluded SNPs with call frequency <98%, minor allele frequency ≤3%, HWE p-value <10^−6^ in controls, heterozygote calls ≥65% and p-value <10^−5^ for a test of differential missingness between cases and controls. We also applied standard QC methods to the samples genotyped on each array and removed samples based on call rate (<98%), mismatch between self-reported gender and genotypic gender, cryptic relatedness between samples, or outlying ethnicity (see Methods S3 and Table S1 for details). After applying these QC filters, our discovery sample contained 1142 cases (98.2%) and 394 controls (95.9%) with data from at least one genotyping array (Illumina 1M-Duo or Affymetrix 6.0). The mean call rate for the discovery samples varied from 99.3% (mouthwash) to 99.5% (blood). For the WTCCC2 data, we removed all samples listed in the sample exclusions file provided by WTCCC. Exclusion criteria included: cryptic relatedness between samples, gender mismatch, low call rate, questionable identity based on previous genotyping, outliers based on heterozygosity, outliers based on principal component plots using HapMap samples, and outliers based on mean A and B intensities on chromosome 22. We also removed all SNPs in the WTCCC2 SNP exclusion files provided based on these criteria: minor allele frequency <1%, information <0.975, call frequency <98% and plate association test p-value <10^−5^. Additionally, we removed SNPs with HWE p-value <10^−6^, which is a more stringent filter than that initially applied by the WTCCC.

The replication samples were genotyped with TaqMan assays (Applied Biosystems, Forest City, CA) using the 7900HT (Duke University Center for Human Genetics, Durham, NC) and OpenArray (HudsonAlpha Institute for Biotechnology, Huntsville, AL) platforms. Genotypes were assigned using ABI's Genotyper software for OpenArray Taqman data, and SDS 2.4 for 7900HT Taqman data. Samples with <90% call rate on the OpenArray platform or poor duplicate concordance were removed. SNPs were removed based on call frequency <90%, discordance between duplicate samples, Mendelian inconsistencies in CEPH trios, and discordance with previously-confirmed genotypes when available.

### Statistical analysis

For the discovery-phase analysis, we combined the markers and genotypes from the Affymetrix 6.0 and Illumina 1M-Duo chips. When a given marker was genotyped and passed QC on both chips, we followed these steps to combine the genotypes: 1) retained Illumina genotypes for symmetric (A/T or C/G) SNPs; 2) performed Fisher's exact test on a 3×2 table of genotype frequencies by chip for each marker, and discarded SNPs with p<0.001; 3) set any remaining discordant genotypes for a given sample to be missing. We combined the Affymetrix 6.0 and Illumina 1M-Duo genotypes for the WTCCC2 samples following the same procedure with one exception: in step 2, due to the much larger sample size, we used a χ^2^ test for different genotype frequencies between the two chips. Finally, we used Fisher's exact test to test for genotype frequency differences between GENEVA controls and WTCCC2 controls, and removed any markers with p-value <0.001.

#### ALS outcome

To test for genetic effects on the primary endpoint of interest, ALS outcome, we restricted our analysis to definite or probable ALS cases who received an initial ALS diagnosis within the 24 months prior to Registry enrollment. We also performed a sensitivity analysis by including other diagnoses (possible ALS and PMA) and/or restricting analysis to cases diagnosed within 12 months of Registry enrollment. Subjects with diagnoses of PLS or PBP and those with longer lags (>24 months) between diagnosis and study consent were excluded from the ALS outcome analysis. Because the number of eligible GENEVA controls was limited at the time of genotyping, we added the publicly-available WTCCC2 controls in an effort to increase the power of our analysis to detect moderate associations with ALS. However, there may be important environmental or other differences between the British non-veteran subjects in the WTCCC2 sample and the US veterans in our study; for this reason, we also performed analyses using the GENEVA controls alone. We excluded all cases and controls with a first-degree family history of ALS (self-reported or, for cases, determined by a Registry neurologist) or a known *SOD1* mutation. Finally, for the purpose of comparison to previously published GWA studies and to match the WTCCC2 controls, we restricted the analysis here to self-reported non-Hispanic Caucasians. After these exclusions, the sample used for ALS outcome analysis included 442 ALS cases, 348 GENEVA controls and 5909 WTCCC2 controls (see Table S2 for the number of samples meeting each exclusion criterion).

Case-control analyses were carried out using unconditional logistic regression, with genotypes coded additively based on the number of copies of the minor allele. We included sex and reference age (age at diagnosis for cases; age at interview for controls) in models using GENEVA controls only. For models including the WTCCC2 controls, we adjusted only for sex, because age data were not available for these subjects at the time of our analysis. For the discovery phase, we also adjusted all models for potential population stratification in our sample by including four components obtained from multi-dimensional scaling analysis (MDS) in PLINK. The MDS components were obtained based on a set of 84 k LD-pruned SNPs discussed in Methods S3. Statistical analyses of the data were carried out using PLINK version 1.07. Code to create Manhattan and QQ plots was adapted from http://gettinggeneticsdone.blogspot.com/2011/04/annotated-manhattan-plots-and-qq-plots.html.

For the replication phase, we performed unconditional logistic regression on the replication samples alone (183 cases, 961 controls) and on the pooled set of samples (replication and discovery) when SNPs were successfully genotyped on both sets of samples. As with the discovery sample, we only considered non-Hispanic Caucasians and restricted analysis to definite or probable ALS cases who received an initial ALS diagnosis within the 24 months prior to study consent. Any genotypes that were discordant between the discovery and replication phases were set to missing for the combined analysis. All p-values reported in the discovery and replication phases are unadjusted for multiple testing.

#### Survival

Our case group for survival analysis included all non-Hispanic Caucasian cases with definite ALS or probable ALS, regardless of the disease duration at the time of consent (n = 639, 29.5% censored). For the survival analysis, we also excluded cases who were already dependent on a ventilator at the time of study enrollment (see Table S2). Using a Cox proportional hazards regression model, we tested for genetic effects on the number of months between diagnosis and either dependence on a ventilator or death, whichever occurred first. We adjusted the model for left-truncation in order to account for the fact that subjects had to survive long enough to contribute a sample to the NRVA DNA bank. All models contained an additive genotype term and included age at diagnosis and sex as covariates. For the discovery phase, the four components from the MDS analysis described above were included as well. We categorized subjects as having bulbar or non-bulbar onset; this variable did not meet the proportional hazards assumption necessary for the model, so we adjusted for site of onset using a stratified model instead of including site of onset as a covariate. Survival analysis was performed using PLINK (v1.06) and R (version 2.10.1), via a PLINK plug-in.

For the replication phase, we performed a survival analysis on the replication samples alone (n = 118, 16.1% censored) and on the pooled set of samples (replication and discovery) when SNPs were successfully genotyped on both sets of samples. As with the discovery sample, we restricted analysis to non-Hispanic Caucasian cases diagnosed with definite or probable ALS.

### Selection of SNPs for replication genotyping

To account for the possibility that ALS-associated SNPs might be enriched in the set of SNPs showing marginal (but not genome-wide) significance, we selected SNPs for replication genotyping using either a strict p-value criterion (p<1.0×10^−6^) or a more lenient p-value criterion (p<1.0×10^−4^) in the presence of other evidence of association (previous independent studies or consistent results across our sensitivity analyses). We examined the genotype cluster plots for SNPs that met either criterion, and only attempted to replicate SNPs that clustered well. In addition to SNPs from the GWAS chips, we decided to genotype additional SNPs in implicated genes/regions, because replication may be observed at the level of a gene rather than a specific SNP. We included common SNPs in coding regions or potential splice sites from 20 selected genes, tagging SNPs from 5 selected genes, and intragenic SNPs in moderate LD (r^2^>0.5) with intergenic SNPs of interest. Based on previously published GWA studies of sporadic ALS, we also genotyped the discovery and replication samples for variation in the genes *DPP6* [7], *ELP3* [3], *ITPR2* [5], and *UNC13A* [9], and for the intergenic SNP rs3849942 on chromosome 9p21.2 [9], [11]. We genotyped the specific implicated SNPs in addition to common coding and possible splicing SNPs in these genes. We also included a SNP in *KIFAP3* previously reported to be associated with survival [8]. The total number of SNPs that were successfully genotyped in the replication samples was 299 (286 selected for the ALS outcome phenotype, 13 selected for survival).

## Results

### Discovery phase

After performing the sample quality control steps described in the Methods, we obtained genotypes on at least one chip for 394 controls (95.9%) and 1142 cases (98.2%). Of these samples, 350 controls (88.8%) and 1104 cases (96.7%) passed QC on both genotyping chips, yielding extremely high-density marker data. After removing cases which did not meet the inclusion criteria for analysis of ALS outcome or survival described above, our final sample included 442 cases and 348 controls for the ALS outcome analysis and 639 cases for the survival analysis. Demographic and clinical characteristics of this discovery sample are shown in Table 1. The veteran cases and controls had similar distributions for military-specific characteristics (military branch with longest service, length of service and deployment to major conflicts) [21]. After SNP quality control and removal of monomorphic SNPs, we obtained genotypes for a total of 1,515,824 autosomal SNP markers (88.1%) from either the Affymetrix 6.0 or Illumina 1M-Duo genotyping chip. 1,280,579 of these markers (84.5%) also met our minimum minor allele frequency (MAF) criterion (≥3%).

**Table 1:** Demographic and clinical characteristics of the discovery and replication samples.

		Discovery cases: ALS outcome	Discovery cases: Survival	Discovery controls	Replication cases: ALS outcome	Replication cases: Survival	Replication controls
**n**		442	639	348	183	118	961
**Male**		435 (98.4%)	627 (98.1%)	331 (95.1%)	115 (62.8%)	80 (67.8%)	755 (78.6%)
**Age (yrs, mean ± SD)**		62.3±10.2	60.4±11.3	62.2±10.7	59.4±12.1	58.8±12.2	63.5±11.5
**Veterans**		442 (100%)	639 (100%)	348 (100%)	8 (4.4%)	12 (10.2%)	396 (41.2%)
**Diagnosis**	**Definite ALS**	115 (26.0%)	200 (31.3%)	-	183 (100%)*	118 (100%)*	-
	**Probable ALS**	327 (74.0%)	439 (68.7%)	-			-
**Time from diagnosis to study consent**	**0–12 months**	293 (66.3%)	285 (44.6%)	-	164 (89.6%)	98 (83.1%)	-
	**12–24 months**	149 (33.7%)	141 (22.1%)	-	19 (10.4%)	16 (13.6%)	-
	**24–36 months**	-	65 (10.2%)	-	-	1 (0.8%)	-
	**>36 months**	-	148 (23.2%)	-	-	3 (2.5%)	-
**Bulbar onset**		114 (25.8%)	130 (20.3%)	-	not available	35 (29.9%)	-
**Median onset to diagnosis (mo)**		11	11	-	not available	9.1	-
**Median survival after diagnosis (mo)**		24.0 (n = 335)	29.0 (n = 442)	-	26.8 (n = 144)	27.1 (n = 99)	-

*Definite/probable ALS breakdown was not available for all replication cases.

**Figure 1:**
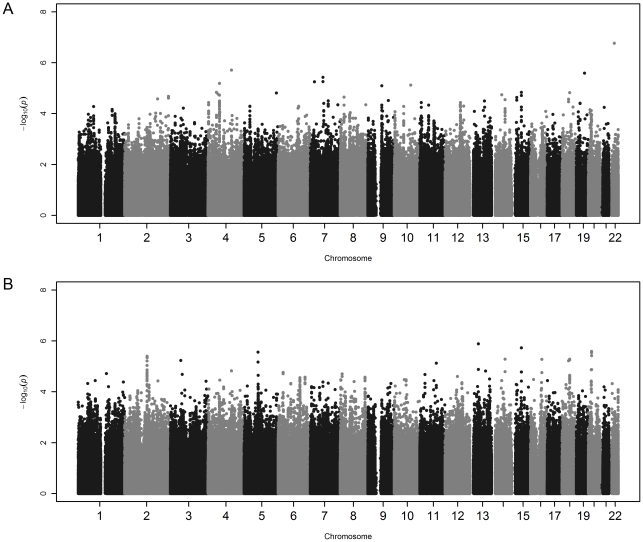
Manhattan plots. a) ALS outcome with GENEVA controls only; b) ALS outcome with GENEVA and WTCCC2 controls included.

**Figure 2:**
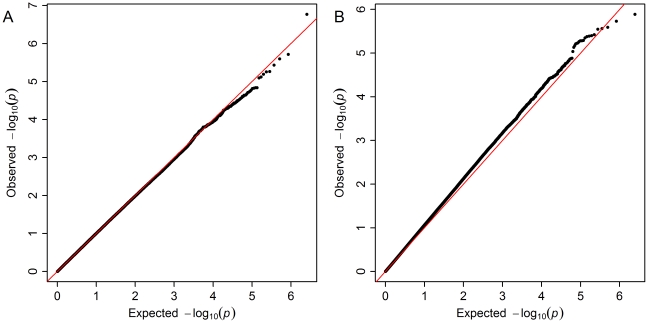
Quantile-quantile plots. a) ALS outcome with GENEVA controls only; b) ALS outcome with GENEVA and WTCCC2 controls included.

Figure 1 shows Manhattan plots for the ALS outcome analysis, both with and without the WTCCC2 controls included. No markers met a genome-wide significance threshold of p<5.0×10^−8^. Detailed results of the most significant 25 SNPs from each analysis are shown in Tables 2 and 3. Two genes, *UNC13C* and *SETBP1*, contain SNPs in the top 25 of both analyses. The QQ plots for the two ALS outcome analyses are shown in Figure 2. For the association analysis including GENEVA controls only, there is a single relatively rare SNP with an observed p-value smaller than expected, rs5762919 in *ZNRF3* (p = 1.7×10^−7^, MAF in cases = 0.04). Unfortunately, this SNP was not successfully genotyped in the WTCCC2 controls, so we were unable to test for a consistent result with the larger set of controls. The QQ plot for the analysis including the WTCCC2 controls deviates from the expected pattern under the null hypothesis of genome-wide absence of association. We examined whether this deviation might be due to residual population stratification by including varying numbers of MDS axes (0, 4, 10, and 20) in the logistic regression models and obtained similar QQ plots even with 20 axes included (data not shown). One potential explanation for this observation might be the age, gender and sample size differences between GENEVA and WTCCC2 controls; another is our inability to adjust for age in the logistic regression models that included the WTCCC2 controls.

**Table 2:** Discovery analysis: SNP association with ALS outcome using GENEVA controls only.

						Minor allele frequency	
Chr	SNP	Pos (build 36)	Closest gene (distance)	Odds ratio (95% CI)	p	Cases	Controls
22	rs5762919	27693344	ZNRF3	0.3 (0.2, 0.5)	1.7E-07	0.04	0.10
4	rs1425419	124785414	LOC285419 (8 kb)	2.1 (1.5, 2.8)	1.9E-06	0.19	0.11
19	rs12327672	43742319	RYR1	0.4 (0.2, 0.5)	2.5E-06	0.04	0.11
7	rs2867161	67274777	STAG3L4 (851 kb)	0.4 (0.3, 0.6)	3.7E-06	0.06	0.13
7	rs17145184	67278550	STAG3L4 (855 kb)	0.4 (0.3, 0.6)	5.4E-06	0.07	0.13
7	rs7777387	22086962	RAPGEF5 (37 kb)	0.3 (0.2, 0.5)	5.6E-06	0.02	0.07
4	rs6816453	60844956	none within 1 Mb	0.5 (0.4, 0.7)	6.4E-06	0.13	0.23
10	rs7912496	87832985	GRID1	0.5 (0.4, 0.7)	7.6E-06	0.11	0.19
9	rs1796991	74507694	TMC1	0.5 (0.4, 0.7)	8.0E-06	0.14	0.23
4	rs7689003	44820582	GNPDA2 (397 kb)	0.6 (0.5, 0.8)	1.5E-05	0.27	0.38
15	rs11071021	52143583	UNC13C	0.6 (0.5, 0.8)	1.5E-05	0.36	0.47
18	rs17783459	40583074	SETBP1	2.1 (1.5, 3.0)	1.5E-05	0.15	0.07
5	rs4868146	171514050	STK10	0.6 (0.5, 0.8)	1.5E-05	0.32	0.42
4	rs4470701	52798649	SPATA18 (140 kb)	0.6 (0.5, 0.8)	1.7E-05	0.25	0.35
14	rs1950202	53909646	CDKN3 (24 kb)	0.6 (0.5, 0.8)	1.8E-05	0.25	0.35
4	rs10050211	60639119	none within 1 Mb	0.5 (0.3, 0.7)	1.9E-05	0.07	0.13
15	rs11071022	52149769	UNC13C	0.6 (0.5, 0.8)	1.9E-05	0.35	0.46
2	rs4972990	231987255	B3GNT7 (13 kb)	0.6 (0.5, 0.8)	2.1E-05	0.36	0.47
8	rs11204102	20137423	LZTS1 (11 kb)	1.7 (1.3, 2.1)	2.2E-05	0.36	0.25
15	rs1563755	27660863	FAM189A1 (11 kb)	0.6 (0.5, 0.8)	2.3E-05	0.28	0.38
15	rs8031323	27662042	FAM189A1 (12 kb)	0.6 (0.5, 0.8)	2.3E-05	0.28	0.38
2	rs2034413	231996393	B3GNT7 (22 kb)	0.6 (0.5, 0.8)	2.4E-05	0.29	0.39
2	rs2034412	231996305	B3GNT7 (22 kb)	0.6 (0.5, 0.8)	2.5E-05	0.29	0.39
2	rs16862162	174441451	SP3 (38 kb)	0.6 (0.5, 0.7)	2.6E-05	0.17	0.27
18	rs9959302	34316183	LOC647946 (725 kb)	0.6 (0.5, 0.8)	2.7E-05	0.31	0.41

**Table 3:** Discovery analysis: SNP association with ALS outcome using GENEVA+WTCCC2 controls.

						Minor allele frequency		
Chr	SNP	Pos (build 36)	Closest gene (distance)	Odds ratio (95% CI)	P	Cases	GENEVA controls	WTCCC2 controls
13	rs9534003	44375190	NUFIP1 (36 kb)	1.7 (1.4, 2.1)	1.3E-06	0.18	0.12	0.12
15	rs11071021	52143583	UNC13C	0.7 (0.6, 0.8)	1.9E-06	0.36	0.47	0.42
20	rs6080544	16987959	PCSK2 (167 kb)	0.7 (0.6, 0.8)	2.6E-06	0.33	0.42	0.39
5	rs7707833	74323619	GCNT4 (35 kb)	0.7 (0.6, 0.8)	2.8E-06	0.33	0.40	0.39
20	rs6075164	16985876	PCSK2 (169 kb)	0.7 (0.6, 0.8)	2.8E-06	0.33	0.43	0.39
20	rs6075165	16986263	PCSK2 (168 kb)	0.7 (0.6, 0.8)	3.8E-06	0.33	0.43	0.39
2	rs6746842	118523065	CCDC93 (35 kb)	1.5 (1.2, 1.7)	4.0E-06	0.35	0.27	0.29
2	rs7571323	118549038	INSIG2 (13 kb)	1.5 (1.2, 1.7)	4.1E-06	0.35	0.27	0.29
2	rs2276695	118531338	INSIG2 (31 kb)	1.4 (1.2, 1.7)	4.2E-06	0.45	0.37	0.38
2	rs11688631	119296905	EN1 (19 kb)	1.5 (1.2, 1.7)	4.5E-06	0.49	0.42	0.45
14	rs6574039	71560768	RGS6	0.6 (0.5, 0.7)	5.1E-06	0.14	0.21	0.18
18	rs639964	40708686	SETBP1	0.7 (0.6, 0.8)	5.2E-06	0.38	0.46	0.44
16	rs12929572	60610083	CDH8	0.7 (0.6, 0.8)	5.2E-06	0.28	0.37	0.35
18	rs617459	40707045	SETBP1	0.7 (0.6, 0.8)	5.5E-06	0.38	0.46	0.44
3	rs2703029	54878655	CACNA2D3	1.4 (1.2, 1.7)	5.8E-06	0.48	0.43	0.45
18	rs7242525	34364676	LOC647946 (676 kb)	0.6 (0.5, 0.7)	6.0E-06	0.13	0.22	0.19
2	rs12466517	118546861	INSIG2 (16 kb)	1.4 (1.2, 1.7)	6.1E-06	0.45	0.37	0.38
5	rs6453104	74324825	GCNT4 (34 kb)	0.7 (0.6, 0.8)	6.7E-06	0.33	0.40	0.39
11	rs688858	87652911	CTSC (13 kb)	1.4 (1.2, 1.7)	7.4E-06	0.40	0.33	0.33
2	rs2161829	118573384	INSIG2	1.4 (1.2, 1.7)	9.2E-06	0.49	0.43	0.42
13	rs6561194	44374808	NUFIP1 (37 kb)	1.5 (1.2, 1.8)	1.3E-05	0.28	0.22	0.23
20	rs6080539	16977494	PCSK2 (177 kb)	0.7 (0.6, 0.8)	1.3E-05	0.30	0.40	0.36
20	rs7272442	15223538	MACROD2	1.4 (1.2, 1.7)	1.4E-05	0.46	0.44	0.48
2	rs6542427	118518151	CCDC93 (30 kb)	1.4 (1.2, 1.7)	1.4E-05	0.33	0.26	0.27
4	rs1425419	124785414	LOC285419 (8 kb)	1.6 (1.3, 1.9)	1.5E-05	0.19	0.11	0.15

**Figure 3:**
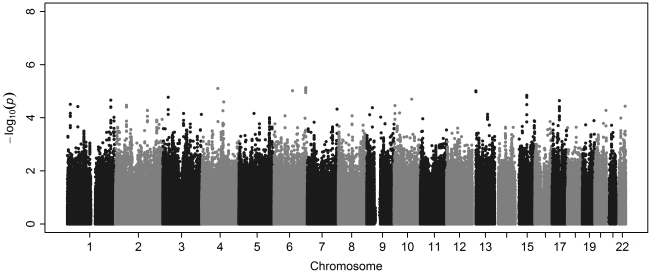
Manhattan plot. Survival analysis of definite/probable ALS cases.

**Figure 4:**
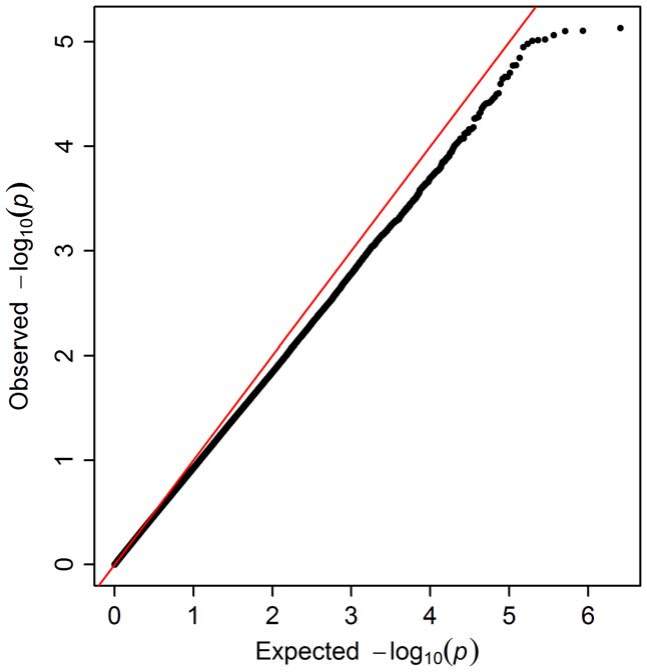
Quantile-quantile plot. Survival analysis of definite/probable ALS cases.

Results from the survival GWAS are shown in Figure 3. Again, no SNPs met a genome-wide significance threshold of p<5.0×10^−8^. The top 25 SNPs from the survival analysis are shown in Table 4. There were five SNPs from the *PARK2* gene in the top ten, all of which are in strong linkage disequilibrium with each other (r^2^>0.85). The QQ plot derived from the survival analysis is shown in Figure 4 and reflects a consistent trend of observed p-values that are larger than expected. This attribute of the QQ plot seems to be related to our exclusion of rare SNPs in the survival analysis. When we included all polymorphic SNPs in the analysis, regardless of MAF, the QQ plot conformed more closely to the pattern expected under the genome-wide absence of association (data not shown).

**Table 4:** Discovery analysis: SNP association with ALS survival time post-diagnosis.

Chr	SNP	pos (build 36)	Closest gene (distance)	Hazard ratio (95% CI)	P	Minor allele frequency
6	rs7740421	162890461	PARK2	0.5 (0.4, 0.7)	7.4E-06	0.08
4	rs6840169	83637534	TMEM150C	1.5 (1.2, 1.7)	7.8E-06	0.16
6	rs7757630	162860228	PARK2	0.5 (0.4, 0.7)	7.9E-06	0.07
6	rs7764218	162854134	PARK2	0.5 (0.4, 0.7)	8.6E-06	0.07
6	rs6904956	162865529	PARK2	0.5 (0.4, 0.7)	9.5E-06	0.08
6	rs564053	94874715	TSG1 (332 kb)	1.4 (1.2, 1.7)	9.6E-06	0.22
13	rs9509608	18196564	ANKRD20A9P (110 kb)	1.6 (1.3, 1.9)	9.7E-06	0.11
13	rs17263670	18162827	ANKRD20A9P (144 kb)	1.6 (1.3, 1.9)	1.0E-05	0.12
6	rs6931162	162863532	PARK2	0.5 (0.4, 0.7)	1.1E-05	0.08
15	rs873961	59135496	RORA	0.7 (0.6, 0.8)	1.4E-05	0.36
15	rs8037669	59137212	RORA	0.7 (0.6, 0.8)	1.7E-05	0.35
3	rs2581182	27899600	EOMES (161 kb)	0.6 (0.5, 0.8)	1.7E-05	0.12
10	rs4933508	91269359	SLC16A12	1.3 (1.2, 1.5)	2.0E-05	0.40
1	rs12024361	223544114	DNAH14	0.7 (0.6, 0.8)	2.2E-05	0.32
1	rs12042076	223544169	DNAH14	0.7 (0.6, 0.8)	2.2E-05	0.32
17	rs12951847	37132640	HAP1	1.5 (1.2, 1.8)	2.3E-05	0.15
4	rs632895	114207962	ANK2	0.6 (0.5, 0.8)	2.5E-05	0.14
1	rs488595	15558384	FHAD1	1.3 (1.2, 1.5)	3.1E-05	0.48
15	rs11632352	59145158	RORA	0.7 (0.6, 0.9)	3.2E-05	0.36
2	rs11885285	56202233	CCDC85A (63 kb)	1.4 (1.2, 1.6)	3.4E-05	0.23
10	rs11252748	4833378	AKR1E2 (25 kb)	1.4 (1.2, 1.6)	3.5E-05	0.22
22	rs9306510	46984975	MIR3201 (64 kb)	1.3 (1.2, 1.6)	3.6E-05	0.34
1	rs618465	54400418	CDCP2 (8 kb)	1.5 (1.2, 1.8)	3.8E-05	0.12
17	rs7213337	37132954	HAP1	1.5 (1.2, 1.8)	3.8E-05	0.14
1	rs10495234	223560206	DNAH14	0.7 (0.6, 0.8)	3.9E-05	0.30

### Replication and pooled analysis

After quality control of the replication samples, we obtained genotypes for 299 SNPs on 278 cases (99.3%) and 1140 controls (98.4%). Of these samples, 183 cases and 961 controls met the inclusion criteria for the analysis of ALS outcome. In addition, 170 cases met the inclusion criteria for analysis of survival, but only 118 of these cases had data for site of onset and could be included in the analysis. The replication SNPs were not all typed on the same platform (two different OpenArray chips were used, along with single-tube TaqMan assays) and quality control was performed separately on each platform. For both ALS outcome and survival, we conducted an analysis using the independent replication samples alone, followed by a pooled analysis of all samples.

The top ten most-significant results from the pooled analysis of ALS outcome are given in Table 5. There is substantial overlap in the results with and without the WTCCC2 controls, as seven markers are in the top ten for both analyses. Although no SNPs met a genome-wide significance threshold in the pooled analysis, three SNPs produced statistically significant results in analysis of the independent replication samples alone after adjustment for multiple testing of 286 SNPs (p<1.75×10^−4^): rs6080539 (177 kb from *PCSK2*), rs7000234 (4 kb from *ZNF704*), and rs3113494 (13 kb from *LOC100506746*) (Table 5). Analogous results for the top ten SNPs in the replication and pooled analysis of the survival outcome are shown in Table 6. None of the SNPs tested in the survival replication analysis showed evidence of association in the replication analysis alone (p>0.05), which might partially be due to the substantially smaller sample size.

**Table 5:** Replication and pooled analysis: SNP association with ALS outcome, including and excluding WTCCC2 controls.

				Discovery analysis		Replication analysis		Pooled analysis	
Chr	SNP	pos (build 36)	Closest gene (distance)	OR (95% CI)	P	OR (95% CI)	p	OR (95% CI)	p
*Without WTCCC2 controls*									
4	rs4833346	127584779	FAT4 (951 kb)	1.5 (1.2, 1.9)	1.2E-04	1.1 (0.8, 1.5)	0.43	1.4 (1.2, 1.6)	2.1E-05
20	rs6075164	16985876	PCSK2 (169 kb)	0.7 (0.5, 0.8)	1.8E-04	0.8 (0.7, 0.9)	0.0016	0.7 (0.6, 0.8)	3.7E-05
20	rs6080539	16977494	PCSK2 (177 kb)	0.6 (0.5, 0.8)	1.1E-04	0.7 (0.6, 0.8)	5.0E-05*	0.7 (0.6, 0.8)	5.1E-05
8	rs7000234	81699669	ZNF704 (4 kb)	2.0 (1.3, 3.1)	8.6E-04	1.8 (1.4, 2.4)	6.1E-05*	1.7 (1.3, 2.3)	1.5E-04
20	rs6080544	16987959	PCSK2 (167 kb)	0.7 (0.5, 0.8)	2.0E-04	1.0 (0.8, 1.4)	0.78	0.7 (0.6, 0.9)	1.9E-04
4	rs3113494	88051625	LOC100506746 (13 kb)	0.7 (0.6, 0.8)	1.9E-04	0.7 (0.6, 0.9)	9.6E-05*	0.8 (0.6, 0.9)	2.0E-04
11	rs2278170	4453422	OR52K1 (13 kb)	0.7 (0.5, 0.8)	5.5E-04	0.7 (0.6, 0.9)	8.0E-04	0.7 (0.6, 0.9)	2.4E-04
6	rs1190270	105731108	POPDC3	0.5 (0.3, 0.7)	1.8E-04	0.6 (0.4, 0.8)	4.0E-04	0.6 (0.4, 0.8)	2.7E-04
8	rs6985069	28142007	ELP3 (37 kb)	0.7 (0.6, 0.9)	6.0E-03	0.7 (0.6, 0.9)	3.3E-04	0.7 (0.6, 0.9)	2.7E-04
11	rs9633905	4453189	OR52K1 (13 kb)	0.7 (0.5, 0.8)	5.9E-04	0.8 (0.6, 0.9)	8.9E-04	0.7 (0.6, 0.9)	4.3E-04
*With WTCCC2 controls*									
4	rs3113494	88051625	LOC100506746 (13 kb)	0.7 (0.6, 0.9)	2.8E-04	0.7 (0.6, 0.9)	9.6E-05*	0.7 (0.6, 0.8)	9.0E-07
11	rs2278170	4453422	OR52K1 (13 kb)	0.8 (0.6, 0.9)	0.0020	0.7 (0.6, 0.9)	8.0E-04	0.7 (0.6, 0.8)	6.7E-06
3	rs17253119	54905813	CACNA2D3	0.6 (0.5, 0.9)	0.0028	0.7 (0.5, 0.9)	0.011	0.6 (0.5, 0.8)	1.8E-05
6	rs1190270	105731108	POPDC3	0.5 (0.3, 0.7)	1.3E-04	0.6 (0.4, 0.8)	4.0E-04	0.6 (0.4, 0.7)	3.6E-05
18	rs7242525	34364676	LOC647946 (676 kb)	0.6 (0.5, 0.7)	6.0E-06	0.7 (0.6, 0.9)	0.0043	0.7 (0.5, 0.8)	1.1E-04
4	rs4833346	127584779	FAT4 (951 kb)	1.4 (1.2, 1.6)	9.8E-05	1.1 (0.8, 1.5)	0.43	1.3 (1.1, 1.5)	1.7E-04
20	rs6080539	16977494	PCSK2 (177 kb)	0.7 (0.6, 0.8)	1.3E-05	0.7 (0.6, 0.8)	5.0E-05*	0.8 (0.7, 0.9)	1.9E-04
8	rs7000234	81699669	ZNF704 (4 kb)	1.7 (1.3, 2.2)	2.4E-04	1.8 (1.4, 2.4)	6.1E-05*	1.5 (1.2, 1.9)	2.2E-04
20	rs6075164	16985876	PCSK2 (169 kb)	0.7 (0.6, 0.8)	2.8E-06	0.8 (0.7, 0.9)	0.0016	0.8 (0.7, 0.9)	2.7E-04
4	rs12651081	88103790	AFF1	0.9 (0.7, 1.0)	0.060	0.8 (0.6, 1.0)	0.10	0.8 (0.7, 0.9)	3.1E-04

* Significant in replication-only analysis after Bonferroni adjustment (p<1.8×10^−4^).

**Table 6:** Replication and pooled analysis: SNP association with ALS survival.

				Discovery analysis		Replication analysis		Pooled analysis	
Chr	SNP	pos (build 36)	Closest gene (distance)	Hazard ratio (95% CI)	P	Hazard ratio (95% CI)	p	Hazard ratio (95% CI)	p
4	rs6840169	83637534	TMEM150C	1.5 (1.2, 1.7)	7.8E-06	1.2 (0.8, 1.9)	0.40	1.4 (1.2, 1.6)	3.9E-05
6	rs564053	94874715	TSG1 (332 kb)	1.4 (1.2, 1.7)	9.6E-06	1.1 (0.7, 1.6)	0.62	1.4 (1.2, 1.6)	5.5E-05
15	rs873961	59135496	RORA	0.7 (0.6, 0.8)	1.4E-05	1.2 (0.9, 1.6)	0.31	0.8 (0.7, 0.9)	7.2E-05
15	rs11632352	59145158	RORA	0.7 (0.6, 0.9)	3.2E-05	1.1 (0.8, 1.5)	0.63	0.8 (0.7, 0.9)	7.5E-05
6	rs7764218	162854134	PARK2	0.5 (0.4, 0.7)	8.6E-06	1.1 (0.7, 1.8)	0.65	0.6 (0.5, 0.8)	1.5E-04
6	rs7757630	162860228	PARK2	0.5 (0.4, 0.7)	7.9E-06	1.1 (0.7, 1.7)	0.72	0.6 (0.5, 0.8)	1.5E-04
15	rs8037669	59137212	RORA	0.7 (0.6, 0.8)	1.7E-05	1.3 (0.9, 1.9)	0.11	0.8 (0.7, 0.9)	1.6E-04
6	rs7740421	162890461	PARK2	0.5 (0.4, 0.7)	7.4E-06	1.2 (0.7, 2)	0.56	0.6 (0.5, 0.8)	2.5E-04
20	rs6027005	57632613	PHACTR3	1.7 (1.3, 2.3)	2.0E-04	0.8 (0.2, 3.3)	0.75	1.7 (1.3, 2.2)	4.1E-04
6	rs6904956	162865529	PARK2	0.5 (0.4, 0.7)	9.5E-06	0.9 (0.7, 1.4)	0.76	0.7 (0.5, 0.8)	5.1E-04

Table 7 shows association results in our dataset for markers and genes implicated in previous GWAS analyses of ALS outcome. For each gene or region of interest, we show not only the marker with the smallest p-value in the pooled analysis, but also the specific SNPs found to be associated with ALS risk in previous studies. We were unable to replicate the previously reported association of the specific SNPs in 9p21.2 and *ITPR2*. SNP rs1541160 in *KIFAP3*, which was implicated by the only other genome-wide analysis of ALS survival [8], also did not show evidence for association in our pooled dataset (HR = 0.95, 95% CI = (0.82, 1.10), p = 0.49). After correcting for multiple testing (based on 57 markers selected from genes previously reported to show association, p<8.8×10^−4^), we detected marginal association with rs6985069 near *ELP3* (p = 2.7×10^−4^ with GENEVA controls only; p = 4.8×10^−4^ with GENEVA+WTCCC2 controls). This is among the top 10 results of the pooled analysis shown in Table 5, but is different from the SNP previously reported as ALS-associated (rs13268593) [3]. Similarly, the specifically-implicated SNP rs10260404 in *DPP6* did not show any evidence for association, while rs17174381 in the same gene had p-values of 0.005 (GENEVA controls only) and 4.4×10^−4^ (GENEVA+WTCCC2 controls) in the pooled analysis.

**Table 7:** Pooled analysis: ALS outcome association with genes implicated by previous GWA studies.

				Pooled analysis (without WTCCC2 controls)		Pooled analysis (with WTCCC2 controls)	
Chr	SNP	pos (build 36)	Gene	OR (95% CI)	P	OR (95% CI)	p
9	rs3849942*	27543281	9p21.2	1.1 (0.9, 1.3)	0.17	1.1 (0.9, 1.2)	0.46
7	rs17174381	153709165	DPP6	2.1 (1.3, 3.5)	0.0051	1.9 (1.3, 2.8)	4.4E-04
7	rs10260404*	153841731	DPP6	1.1 (0.9, 1.2)	0.47	1.0 (0.9, 1.1)	0.97
8	rs6985069	28142007	ELP3	0.7 (0.6, 0.9)	2.7E-04	0.8 (0.7, 0.9)	4.8E-04
8	rs13268953*	28111155	ELP3	1.0 (0.8, 1.1)	0.83	1.0 (0.9, 1.1)	0.97
12	rs11048556	26559935	ITPR2	1.3 (0.9, 1.7)	0.14	1.4 (1.1, 1.9)	0.0068
12	rs2306677*	26527653	ITPR2	1.0 (0.7, 1.3)	0.84	0.9 (0.7, 1.1)	0.25
19	rs3746200	17573374	UNC13A	0.8 (0.6, 1.0)	0.050	0.7 (0.6, 0.9)	0.0068
19	rs12608932* +	17613689	UNC13A	1.2 (1.0, 1.5)	0.039	1.2 (1.0, 1.4)	0.046

*Associated SNPs from previous GWAS.

+ Results from discovery phase only (SNP failed in replication).

## Discussion

Our GWAS of US veterans did not identify any genetic associations that reached genome-wide significance (p<5.0×10^−8^) for either ALS outcome or survival. After following up a smaller number of SNPs, three SNPs were significantly associated with ALS outcome in a replication-only analysis after adjusting for multiple testing. Two of these markers fall outside the transcriptional boundaries of their closest genes (*PCSK2* and *ZNF704*), and one is 13 kb from a hypothetical non-coding RNA (*LOC100506746*). Several other SNPs with originally similar or stronger associations (e.g. rs11071021 from *UNC13C*, which was in the top 25 SNPs for the ALS outcome analysis with or without WTCCC2 controls) did not meet criteria for statistical significance in the replication phase.

Several factors may have contributed to this lack of intra-study replication. Perhaps most importantly, veterans comprised all of the cases and controls in our discovery phase analysis, but comprised only 4% of the cases and 41% of the controls in our replication sample. This also created a substantial difference in gender distribution between the two samples. Because the higher rate of ALS reported for US veterans may be partially due to military-related environmental exposures and corresponding gene-by-environment interactions, the heterogeneity in environmental exposures between the discovery and replication samples could be partly responsible for the lack of replication. In this case, a much larger veteran-only replication sample would be required to detect such interactions. Study design differences may also play a role: the cases in the discovery study were derived from a registry-based sample that included incident and prevalent cases, while the cases in the replication study were derived from a clinic- or population-based sample and were thus enriched for incident cases. Therefore, patients analyzed in the discovery phase were, on average, enrolled longer after diagnosis than patients analyzed in the replication phase (Table 1), and patients who died within two years of diagnosis may be under-represented in the discovery sample. We accommodated this in our analysis by restricting the ALS outcome analysis to incident cases and by adjusting the survival analysis for the length-biased nature of the data. As shown in Table 1, this phenotypic restriction led to similar observed survival characteristics of the two groups of patients (median survival 24.0 vs. 26.8 months for the patients included in the ALS outcome analysis; 29.0 vs. 27.1 months for the patients included in the survival analysis). In addition to the study design differences, it is possible that diagnostic heterogeneity also contributes to the difficulty of intra-study replication. The clinical criteria used by the NRVA neurologists were carefully standardized and cross-validated to minimize diagnostic heterogeneity within the discovery sample. Such standardization was not possible in our replication dataset, because these samples were independently ascertained.

While none of the SNPs evaluated in this study reached genome-wide significance, we noted several interesting genes as potential candidates for further study. For example, results from our discovery phase ALS outcome analysis indicated weakly suggestive association with *UNC13C* which, like the previously implicated *UNC13A*, is a homolog of the *C. elegans* gene *UNC-13.* Both of these genes code for proteins with neurological effects. Although not replicated, our discovery phase survival analysis suggested a marginal association with variants in the *PARK2* gene; mutations in *PARK2* are known to cause Parkinson's disease [42], [43]. Finally, *PCSK2* is an interesting candidate gene given recent work on the potential relationship between ALS and metabolic phenotypes such as hyperlipidemia, BMI and type 2 diabetes [44], [45]. SNPs in *PCSK2* have been shown to be associated with type 2 diabetes in several ethnic populations [46]–[48]. We again note that none of these genes contained markers that were significant at a genome-wide level during the discovery phase of this analysis, and present these genes only as plausible candidates for study in independent populations.

Putting our GWAS in the context of previously published genome-wide studies, we were unable to conclusively replicate the previously-reported associations between ALS and *ITPR2*, *UNC13A* or the 9p21.2 locus. However, at the level of the gene rather than the specific previously implicated SNP, we observed some evidence of association for *ELP3* and *DPP6*. A previous genome-wide survival analysis identified a SNP in *KIFAP3* as associated with increased survival of ALS patients. Our study failed to replicate this association, consistent with another independent study [49]. We note that, although our study is one of the larger ALS genome-wide screens performed to date, it is still underpowered to detect common variants with modest effects on such a rare disease. For example, it would require approximately 2450 cases and an equal number of controls to have 80% power to detect the magnitude of the effect of rs10260404 previously described in the *DPP6* gene [7].

Perhaps the most important factor contributing to the difficulty of replication in independent populations is the underlying genetic heterogeneity of the disease. Linkage studies of familial ALS have implicated twelve loci and eight genes [50]. In addition to this locus heterogeneity, there is also extensive allelic heterogeneity; within the *SOD1* gene, more than 125 non-synonymous coding changes have been described [50]. If similar genetic heterogeneity exists in sporadic ALS, meta-analysis of many association studies will be needed to generate the very large sample sizes required to reliably identify causative variants. Although results from a number of GWA studies in ALS have not been successfully replicated, two recent studies did replicate an association with a common hexanucleotide repeat in the 9p21 region that accounts for a large proportion of familial and sporadic disease in European and North American populations [17], [18]. This region was initially identified through a genome-wide screen [9]. Although a risk haplotype containing expanded repeats in this region is well-tagged by a particular SNP (rs3849942), the association signal from analysis of the repeats is much stronger than the association signal from the SNP [17], which underscores the difficulty of identifying causal variants other than SNPs using chip-based GWAS analysis. Given the known genetic heterogeneity in ALS and the possibility that multiple rare, highly-penetrant variants may account for a greater proportion of currently unexplained disease than common variants with lower penetrance, exome or whole genome sequencing may prove to be more successful than GWAS studies in revealing the genetic underpinnings of this devastating disease.

## Supporting Information

Methods S1 **Details of the replication genotyping samples.**
(DOC)

Methods S2 **Details of genotyping and SNP QC procedures.**
(DOC)

Methods S3 **GWAS sample QC procedures.**
(DOC)

Table S1 **Quality control of discovery samples.**
(DOC)

Table S2 **Samples excluded from discovery phase.**
(DOC)

## References

[1] Logroscino G, Traynor BJ, Hardiman O, Chio A, Mitchell D (2010). Incidence of amyotrophic lateral sclerosis in Europe.. J Neurol Neurosurg Psychiatry.

[2] Byrne S, Walsh C, Lynch C, Bede P, Elamin M (2011). Rate of familial amyotrophic lateral sclerosis: a systematic review and meta-analysis.. J Neurol Neurosurg Psychiatry.

[3] Simpson CL, Lemmens R, Miskiewicz K, Broom WJ, Hansen VK (2009). Variants of the elongator protein 3 (ELP3) gene are associated with motor neuron degeneration.. Hum Mol Genet.

[4] Dunckley T, Huentelman MJ, Craig DW, Pearson JV, Szelinger S (2007). Whole-genome analysis of sporadic amyotrophic lateral sclerosis.. N Engl J Med.

[5] van Es MA, Van Vught PW, Blauw HM, Franke L, Saris CG (2007). ITPR2 as a susceptibility gene in sporadic amyotrophic lateral sclerosis: a genome-wide association study.. Lancet Neurol.

[6] Cronin S, Berger S, Ding J, Schymick JC, Washecka N (2008). A genome-wide association study of sporadic ALS in a homogenous Irish population.. Hum Mol Genet.

[7] van Es MA, van Vught PW, Blauw HM, Franke L, Saris CG (2008). Genetic variation in DPP6 is associated with susceptibility to amyotrophic lateral sclerosis.. Nat Genet.

[8] Landers JE, Melki J, Meininger V, Glass JD, van den Berg LH (2009). Reduced expression of the Kinesin-Associated Protein 3 (KIFAP3) gene increases survival in sporadic amyotrophic lateral sclerosis.. Proc Natl Acad Sci U S A.

[9] van Es MA, Veldink JH, Saris CG, Blauw HM, van Vught PW (2009). Genome-wide association study identifies 19p13.3 (UNC13A) and 9p21.2 as susceptibility loci for sporadic amyotrophic lateral sclerosis.. Nat Genet.

[10] Laaksovirta H, Peuralinna T, Schymick JC, Scholz SW, Lai SL (2010). Chromosome 9p21 in amyotrophic lateral sclerosis in Finland: a genome-wide association study.. Lancet Neurol.

[11] Shatunov A, Mok K, Newhouse S, Weale ME, Smith B (2010). Chromosome 9p21 in sporadic amyotrophic lateral sclerosis in the UK and seven other countries: a genome-wide association study.. Lancet Neurol.

[12] Cronin S, Tomik B, Bradley DG, Slowik A, Hardiman O (2009). Screening for replication of genome-wide SNP associations in sporadic ALS.. Eur J Hum Genet.

[13] Schymick JC, Scholz SW, Fung HC, Britton A, Arepalli S (2007). Genome-wide genotyping in amyotrophic lateral sclerosis and neurologically normal controls: first stage analysis and public release of data.. Lancet Neurol.

[14] Chio A, Schymick JC, Restagno G, Scholz SW, Lombardo F (2009). A two-stage genome-wide association study of sporadic amyotrophic lateral sclerosis.. Hum Mol Genet.

[15] Van Es MA, Van Vught PW, Veldink JH, Andersen PM, Birve A (2009). Analysis of FGGY as a risk factor for sporadic amyotrophic lateral sclerosis.. Amyotroph Lateral Scler.

[16] Fernandez-Santiago R, Sharma M, Berg D, Illig T, Anneser J (2011). No evidence of association of FLJ10986 and ITPR2 with ALS in a large German cohort.. Neurobiol Aging.

[17] Renton AE, Majounie E, Waite A, Simon-Sanchez J, Rollinson S (2011). A Hexanucleotide Repeat Expansion in C9ORF72 Is the Cause of Chromosome 9p21-Linked ALS-FTD.. Neuron.

[18] Dejesus-Hernandez M, Mackenzie IR, Boeve BF, Boxer AL, Baker M (2011). Expanded GGGGCC Hexanucleotide Repeat in Noncoding Region of C9ORF72 Causes Chromosome 9p-Linked FTD and ALS.. Neuron.

[19] Wang H, O'Reilly EJ, Weisskopf MG, Logroscino G, McCullough ML (2011). Smoking and risk of amyotrophic lateral sclerosis: a pooled analysis of 5 prospective cohorts.. Arch Neurol.

[20] Gallo V, Bueno-De-Mesquita HB, Vermeulen R, Andersen PM, Kyrozis A (2009). Smoking and risk for amyotrophic lateral sclerosis: analysis of the EPIC cohort.. Ann Neurol.

[21] Schmidt S, Kwee LC, Allen KD, Oddone EZ (2010). Association of ALS with head injury, cigarette smoking and APOE genotypes.. J Neurol Sci.

[22] Chen H, Richard M, Sandler DP, Umbach DM, Kamel F (2007). Head injury and amyotrophic lateral sclerosis.. Am J Epidemiol.

[23] Binazzi A, Belli S, Uccelli R, Desiato MT, Talamanca IF (2009). An exploratory case-control study on spinal and bulbar forms of amyotrophic lateral sclerosis in the province of Rome.. Amyotroph Lateral Scler.

[24] Fang F, Kwee LC, Allen KD, Umbach DM, Ye W (2010). Association between blood lead and the risk of amyotrophic lateral sclerosis.. Am J Epidemiol.

[25] Campbell AM, Williams ER, Barltrop D (1970). Motor neurone disease and exposure to lead.. J Neurol Neurosurg Psychiatry.

[26] Kamel F, Umbach DM, Hu H, Munsat TL, Shefner JM (2005). Lead exposure as a risk factor for amyotrophic lateral sclerosis.. Neurodegener Dis.

[27] Gresham LS, Molgaard CA, Golbeck AL, Smith R (1986). Amyotrophic lateral sclerosis and occupational heavy metal exposure: a case-control study.. Neuroepidemiology.

[28] Bonvicini F, Marcello N, Mandrioli J, Pietrini V, Vinceti M (2010). Exposure to pesticides and risk of amyotrophic lateral sclerosis: a population-based case-control study.. Ann Ist Super Sanita.

[29] Sutedja NA, Veldink JH, Fischer K, Kromhout H, Heederik D (2009). Exposure to chemicals and metals and risk of amyotrophic lateral sclerosis: a systematic review.. Amyotroph Lateral Scler.

[30] Mattsson P, Lonnstedt I, Nygren I, Askmark H (2010). Physical fitness, but not muscle strength, is a risk factor for death in amyotrophic lateral sclerosis at an early age.. J Neurol Neurosurg Psychiatry.

[31] Beghi E, Logroscino G, Chio A, Hardiman O, Millul A (2010). Amyotrophic lateral sclerosis, physical exercise, trauma and sports: results of a population-based pilot case-control study.. Amyotroph Lateral Scler.

[32] Horner RD, Grambow SC, Coffman CJ, Lindquist JH, Oddone EZ (2008). Amyotrophic lateral sclerosis among 1991 Gulf War veterans: evidence for a time-limited outbreak.. Neuroepidemiology.

[33] Haley RW (2003). Excess incidence of ALS in young Gulf War veterans.. Neurology.

[34] Horner RD, Kamins KG, Feussner JR, Grambow SC, Hoff-Lindquist J (2003). Occurrence of amyotrophic lateral sclerosis among Gulf War veterans.. Neurology.

[35] Weisskopf MG, O'Reilly EJ, McCullough ML, Calle EE, Thun MJ (2005). Prospective study of military service and mortality from ALS.. Neurology.

[36] Allen KD, Kasarskis EJ, Bedlack RS, Rozear MP, Morgenlander JC (2008). The National Registry of Veterans with amyotrophic lateral sclerosis.. Neuroepidemiology.

[37] Brooks BR, Miller RG, Swash M, Munsat TL (2000). El Escorial revisited: revised criteria for the diagnosis of amyotrophic lateral sclerosis.. Amyotroph Lateral Scler Other Motor Neuron Disord.

[38] Brooks BR (1994). El Escorial World Federation of Neurology criteria for the diagnosis of amyotrophic lateral sclerosis. Subcommittee on Motor Neuron Diseases/Amyotrophic Lateral Sclerosis of the World Federation of Neurology Research Group on Neuromuscular Diseases and the El Escorial “Clinical limits of amyotrophic lateral sclerosis” workshop contributors.. J Neurol Sci.

[39] Schmidt S, Allen KD, Loiacono VT, Norman B, Stanwyck CL (2008). Genes and Environmental Exposures in Veterans with Amyotrophic Lateral Sclerosis: the GENEVA study. Rationale, study design and demographic characteristics.. Neuroepidemiology.

[40] Consortium WTCC (2007). Genome-wide association study of 14,000 cases of seven common diseases and 3,000 shared controls.. Nature.

[41] McCarthy MI, Abecasis GR, Cardon LR, Goldstein DB, Little J (2008). Genome-wide association studies for complex traits: consensus, uncertainty and challenges.. Nat Rev Genet.

[42] Kitada T, Asakawa S, Hattori N, Matsumine H, Yamamura Y (1998). Mutations in the parkin gene cause autosomal recessive juvenile parkinsonism.. Nature.

[43] Mata IF, Lockhart PJ, Farrer MJ (2004). Parkin genetics: one model for Parkinson's disease.. Hum Mol Genet.

[44] Sutedja NA, van der Schouw YT, Fischer K, Sizoo EM, Huisman MH (2011). Beneficial vascular risk profile is associated with amyotrophic lateral sclerosis.. J Neurol Neurosurg Psychiatry.

[45] Jawaid A, Murthy SB, Wilson AM, Qureshi SU, Amro MJ (2010). A decrease in body mass index is associated with faster progression of motor symptoms and shorter survival in ALS.. Amyotroph Lateral Scler.

[46] Zheng X, Ren W, Zhang S, Liu J, Li S (2011). Association of type 2 diabetes susceptibility genes (TCF7L2, SLC30A8, PCSK1 and PCSK2) and proinsulin conversion in a Chinese population.. Mol Biol Rep.

[47] Leak TS, Keene KL, Langefeld CD, Gallagher CJ, Mychaleckyj JC (2007). Association of the proprotein convertase subtilisin/kexin-type 2 (PCSK2) gene with type 2 diabetes in an African American population.. Mol Genet Metab.

[48] Yoshida H, Ohagi S, Sanke T, Furuta H, Furuta M (1995). Association of the prohormone convertase 2 gene (PCSK2) on chromosome 20 with NIDDM in Japanese subjects.. Diabetes.

[49] Traynor BJ, Nalls M, Lai SL, Gibbs RJ, Schymick JC (2010). Kinesin-associated protein 3 (KIFAP3) has no effect on survival in a population-based cohort of ALS patients.. Proc Natl Acad Sci U S A.

[50] Dion PA, Daoud H, Rouleau GA (2009). Genetics of motor neuron disorders: new insights into pathogenic mechanisms.. Nat Rev Genet.

