# Supplementary material for: A High-Density Genome-Wide Association Screen of Sporadic ALS in US Veterans
Source: PLoS One. 2012 Mar 28;7(3):e32768. doi: 10.1371/journal.pone.0032768 (PMC3314660; doi:10.1371/journal.pone.0032768)
Supplement: Methods S1 — Details of the replication genotyping samples. (DOC) [file pone.0032768.s001.doc]

# Supplementary Methods S1. Details of the replication genotyping samples

***Northeast ALS (NEALS) Consortium***: Volunteers were recruited and enrolled at 30 medical centers around the U.S. participating in the Multicenter Study for the Validation of ALS Biomarkers trial. Volunteers were recruited through neurology and ALS specialty clinics, using brochures and mass mailings to neurologists and patients, as well as links on various ALS and other neurodegenerative-related websites. Following informed consent and eligibility review, volunteers between the ages of 30 and 80 years were enrolled and whole blood was collected. Cases were diagnosed with ALS according to the El Escorial criteria of definite, probable, laboratory-supported probable, or possible or suspected ALS [1]. The controls used in this study had no known neurodegenerative diagnosis.

***Agricultural Health Study (AHS)***: Subjects were private pesticide applicators and their spouses who were initially recruited as part of the Farming and Movement Evaluation (FAME) study, a case-control study of Parkinson’s disease (PD) nested within the AHS, described elsewhere [2]. The controls were selected using stratified random sampling of all AHS subjects and were frequency-matched to FAME cases by age, sex, and state of residence. All controls were assessed in person by either a neurologist or neurology technician and were excluded if they were ultimately diagnosed with PD or parkinsonism, cognitively impaired or seriously ill. Whole blood was collected from all participating subjects.

***New England Study of ALS***: Recruitment details and study design for the New England study of ALS are given elsewhere [3]. Volunteers with ALS were recruited between 1993 and 1996 from the Neuromuscular Research Unit at New England Medical Center and the Neurophysiology Laboratory at Brigham and Women’s Hospital. Population controls were identified using random telephone screening and were frequency-matched to cases based on age bins and telephone area codes. Whole blood samples were collected from a subset of enrolled cases and controls at a central laboratory. Both cases and controls were eligible if they lived in New England at least 50% of the year, spoke English and were mentally competent. Cases were required to have had an initial ALS diagnosis within the previous two years. Controls were excluded if they lived more than 20 miles from the sample-collection facility; had received a physician diagnosis of Alzheimer’s disease or other dementia, Parkinson’s disease or parkinsonism, ALS or other motor neuron diseases, neuropathy, or post-polio syndrome.

***Genetics and Epidemiology of Motor Neuron disease (GEM)***: The GEM case-control study of ALS was carried out from 1996 through 2000 at Kaiser Permanente Northern California (KPNC), a large health maintenance program in northern California. Study design and methodological details for this study are described elsewhere [4]. Study subjects were recruited from the KPNC membership in a 22-county region in northern California. Potential cases of ALS were identified through physician referrals and through active surveillance of KPNC computerized outpatient, inpatient, and pharmacy databases. In order to apply a modified version of the El Escorial criteria developed by the World Federation of Neurology Research Committee Group on Motor Neuron Diseases [1], medical records of potential cases were reviewed by the study neurologist (CMT) who used information from neurologic examinations and clinical history, laboratory studies, electrophysiologic tests, and imaging studies. Eligible patients included those who were 18 years or older, and newly diagnosed with ALS between 1 January 1996 and 31 March 2000. Of the 193 eligible cases, 166 (86%) agreed to participate in the case-control study. Control subjects were randomly selected from computerized KPMCP membership records and frequency-matched to cases by sex and birth year; twice as many controls as cases were selected. Of the 610 eligible controls invited to participate in the study, 389 (64%) agreed to participate. To obtain information about risk factors for ALS, all subjects completed an in-person structured interview. Whole blood samples were obtained for more than 85% of subjects for DNA extraction and genotyping.

# References

1. Brooks BR, Miller RG, Swash M, Munsat TL (2000) El Escorial revisited: revised criteria for the diagnosis of amyotrophic lateral sclerosis. Amyotroph Lateral Scler Other Motor Neuron Disord 1: 293-299.

2. Tanner CM, Kamel F, Ross GW, Hoppin JA, Goldman SM, et al. (2011) Rotenone, paraquat, and Parkinson's disease. Environ Health Perspect 119: 866-872.

3. Kamel F, Umbach DM, Munsat TL, Shefner JM, Hu H, et al. (2002) Lead exposure and amyotrophic lateral sclerosis. Epidemiology 13: 311-319.

4. Popat RA, Tanner CM, van den Eeden SK, Bernstein AL, Bloch DA, et al. (2007) Effect of non-steroidal anti-inflammatory medications on the risk of amyotrophic lateral sclerosis. Amyotroph Lateral Scler 8: 157-163.
