# Supplementary material for: A High-Density Genome-Wide Association Screen of Sporadic ALS in US Veterans
Source: PLoS One. 2012 Mar 28;7(3):e32768. doi: 10.1371/journal.pone.0032768 (PMC3314660; doi:10.1371/journal.pone.0032768)
Supplement: Methods S2 — Details of genotyping and SNP QC procedures. (DOC) [file pone.0032768.s002.doc]

# Supplementary Methods S2. Details of genotyping and SNP QC procedures

## Affymetrix 6.0 chip

Genotypes were assigned using Affymetrix Power Tools software (version 1.10.2) with Birdseed v2 models and a confidence score cutoff of 0.1. For genotype calling, the samples were split into three equal batches, which were balanced for affection status, gender and race. We checked for batch genotyping effects by performing a chi-square test for different allele frequencies per marker across the three groups. The overall group of samples only included 45 females, so each batch was padded with a common set of high-quality female study samples to ensure the presence of at least 30 female samples in each group, which is recommended by Affymetrix for proper performance of the Birdseed algorithm. We checked these common female samples for genotype concordance across the batches as an extra test for batch genotyping effects, and then retained a single set of genotypes for each female study sample to use in analysis.

## Illumina 1M chip

Genotypes were called using Illumina’s GenomeStudio software and a cluster file was developed using the study samples as described below. Samples were initially genotyped using the standard cluster file from Illumina, which is based on HapMap samples. Using these clusters as a starting point, we then undertook an iterative genotyping/cluster QC process in order to create a new cluster file based on the highest-quality samples in our specific dataset.

As an overview, we divided the bulk of our samples into three large groups, and removed all samples with initial call rates < 98%. We applied a sequence of filters (steps 1-7 below) to the first batch of samples (project 1) and created a GenomeStudio cluster file with the resulting markers and genotype clusters. Then, we loaded the samples from project 2 into GenomeStudio and clustered these using the file from project 1. All markers with initial call rate <98% in project 2 were removed and a project 2 cluster file was generated. This cluster file contained all of the same cluster areas for called markers as in project 1; the only difference was the markers removed due to low call rate in project 2. We then repeated the process (removing markers with call rate < 98%) for our final large batch of samples in project 3. The project 3 cluster file was the final cluster file used for genotyping and was then applied to all samples genotyped on the Illumina 1M chip.

The sequence of filters to remove problematic SNPs was based on the following metrics available in GenomeStudio:

- AB R mean. Mean normalized intensity (R) for the heterozygote cluster
- AB T mean. Mean normalized theta value (T) for the heterozygote cluster
- Cluster separation (range: 0 to 1). Measure of the separation between clusters in the theta dimension
- Het excess (range: -1 to 1). Measure of excess heterozygote calls relative to the expectation under Hardy-Weinberg equilibrium: takes a value of -1 when no heterozygotes are called and a value of 1 when all genotypes are called heterozygotes
- R dev. Measure of the spread of normalized intensity for a given genotype cluster
- T dev. Measure of the spread of normalized theta for a given genotype cluster.

The goal of the filtering described here was to eliminate most poorly-clustering markers while retaining as many good markers as possible. To this end, we employed a combination of cutoffs for QC metrics with visual inspection of cluster plots for markers at the boundaries of the cutoffs. If the intensity cluster plots near the boundary looked problematic (>3 genotype clusters, poorly-defined clusters, etc.), we adjusted the cutoff to remove additional markers.

The QC filtering steps we employed were:

1. Low-intensity markers: removed markers with AB R mean <= 0.25.
2. Heterozygote clusters shifted too close to a homozygote cluster: removed markers with AB T mean < 0.2 or ≥ 0.8. Visually inspected the plots for markers on the boundaries (0.2 ≤ AB T mean ≤ 0.25; 0.75 ≤ AB T mean < 0.8) and determined appropriate cutoff.
3. Poor separation on theta axis: removed markers with cluster separation < 0.3.
4. Visually reviewed markers with cluster separation between 0.3-0.32; removed these as appropriate.
5. More heterozygotes called than expected under HWE: removed markers with het excess ≥ 0.2.
6. Fewer heterozygotes called than expected under HWE: visually inspected a subset of intensity plots for polymorphic markers with het excess ≤ -0.3. This was a prohibitively large number of markers to inspect exhaustively, so we prioritized our visual inspection as follows to remove markers with >3 called genotype clusters:
   1. Markers with R dev >= .05 for the minor-allele homozygote: removed markers with multiple minor-allele homozygote clusters (e.g. 2 distinct clusters in the R dimension called together as homozygotes).
   2. Wide homozygote clusters: inspected markers with AA or BB T Dev >= 0.05, and removed markers with multiple clusters on the T axis that were called together as homozygotes.
   3. Wide heterozygote cluster: inspected a subset of markers with AB T Dev >=.05. We prioritized markers with tight homozygote clusters (as measured by AA T Dev and BB T Dev) and a wide heterozygote cluster, and visually determined a boundary for AB T Dev that corresponded to markers with a single heterozygote cluster.
7. Replication errors: removed any remaining snps with >3 replication errors.
8. Call rate: removed all remaining markers with call rate < 98%.

We checked for batch genotyping effects by performing a chi-square test for different allele frequencies per marker across the three groups. Because this analysis was based on autosomal SNPs, we have not described QC steps applied to mitochondrial, X- or Y-chromosome SNPs, or intensity-only markers on the Illumina 1M chip.
