# Supplementary material for: A High-Density Genome-Wide Association Screen of Sporadic ALS in US Veterans
Source: PLoS One. 2012 Mar 28;7(3):e32768. doi: 10.1371/journal.pone.0032768 (PMC3314660; doi:10.1371/journal.pone.0032768)
Supplement: Methods S3 — GWAS sample QC procedures. (DOC) [file pone.0032768.s003.doc]

# Supplementary Methods S3. GWAS sample QC procedures

We selected a set of SNPs in linkage equilibrium for use in cryptic relatedness and ethnicity checks. We began by considering SNPs common to both the Illumina and Affymetrix chips, with minor allele frequency > 5% in non-Hispanic whites, HWE p-value > .001 in non-Hispanic white controls, and satisfactory genotypes on both chips in our data. We used PLINK’s LD-pruning capability to remove SNPs in short-range LD with each other based on the variance inflation factor (VIF >2), calculated on windows of 50 SNPs at a time, with a step size of 5 SNPs. We then repeated the LD-pruning step on the resulting SNPs to remove remaining regions of long-range LD, using a cutoff of r^2^ > 0.2, windows of 500 SNPs and a step size of 50 SNPs. After these filters were applied, 83,718 SNPs remained. We refer to these as the set of 84k LD-pruned SNPs. We tested all pairs of samples for cryptic relatedness using this set of SNPs in linkage equilibrium and arbitrarily removed one sample of each pair with estimated IBD sharing > 0.15. Finally, we checked for ethnicity outliers using multidimensional scaling plots of the LD-pruned set of SNPs.
