# Supplementary material for: A High-Density Genome-Wide Association Screen of Sporadic ALS in US Veterans
Source: PLoS One. 2012 Mar 28;7(3):e32768. doi: 10.1371/journal.pone.0032768 (PMC3314660; doi:10.1371/journal.pone.0032768)
Supplement: Table S1 — Quality control of discovery samples. (DOC) [file pone.0032768.s004.doc]

# Table S1. Quality control of discovery samples

|  | **Cases (blood)** | **Cases (mouthwash)** | **Cases (total)** | **Controls** |
| --- | --- | --- | --- | --- |
| **Samples eligible for genotyping** | 987 | 176 | 1163 | 411 |
| Whole-genome amplified | 1 (0.1%) | 16 (9.1%) | 17 (1.5%) | 0 |
| **Samples removed during QC** |  |  |  |  |
| Genotyping failed | 3 (0.3%) | 1 (0.6%) | 4 (0.3%) | 12 (2.9%) |
| Gender mismatch | 3 (0.3%) | 2 (1.1%) | 5 (0.4%) | 2 (0.5%) |
| Low call rate | 2 (0.2%) | 2 (1.1%)* | 4 (0.3%) | 1 (0.2%) |
| Cryptic relatedness | 3 (0.3%) | 2 (1.1%) | 5 (0.4%) | 0 |
| Outlying ethnicity | 1 (0.1%) | 1 (0.6%) | 2 (0.2%) | 2 (0.5%) |
| Updated diagnosis: no ALS | 1 (0.1%) | 0 (0%) | 1 (0.1%) | - |
| **Total samples post-QC** | 974 (98.7%) | 168 (95.5%) | 1142 (98.2%) | 394 (95.9%) |
| **Mean genotype call rate (SD)** | 0.995 (0.0014) | 0.993 (0.0039) | 0.995 (0.0021) | 0.994 (0.0024) |
| **Cross-platform genotype concordance** | 0.999 | 0.998 | 0.999 | 0.998 |

*One mouthwash-amplified sample was removed for low call rate; all other samples removed during QC were non-amplified.
