# Supplementary material for: A High-Density Genome-Wide Association Screen of Sporadic ALS in US Veterans
Source: PLoS One. 2012 Mar 28;7(3):e32768. doi: 10.1371/journal.pone.0032768 (PMC3314660; doi:10.1371/journal.pone.0032768)
Supplement: Table S2 — Samples excluded from discovery phase. (DOC) [file pone.0032768.s005.doc]

# Table S2. Samples excluded from discovery phase

|  | **Cases** | **Controls** |
| --- | --- | --- |
| **Samples eligible for analysis post-QC** | 1142 | 394 |
| Race/ethnicity: non-Caucasian^1,2^ | 106 (9.3%) | 46 (11.7%) |
| Incidence status: >24 months between diagnosis with definite/probable ALS and study enrollment^1^ | 274 (24.0%) | - |
| Diagnosis: probable ALS or PMA^1,2^ | 270 (23.6%) | - |
| Diagnosis: PLS or PBP^1,2^ | 96 (8.4%) | - |
| Known SOD1 mutation or 1st degree family history of ALS^1,2^ | 25 (2.2%) | 0 |
| Dependent on a ventilator at study entry^2^ | 51 (4.5%) | - |

^1^Removed from analysis of ALS outcome

^2^Removed from survival analysis

Cells are not mutually exclusive, i.e. one sample might be counted in multiple cells
